# Dynamic Changes in Antibodies and Proteome in Breast Milk of Mothers Infected with Wild-Type SARS-CoV-2 and Omicron: A Longitudinal Study

**DOI:** 10.3390/nu17081396

**Published:** 2025-04-21

**Authors:** Yaqiong Guo, Cheng Li, Minjie Tan, Yuexiao Chen, Shuai Zhu, Cheng Zhi, Jing Zhu

**Affiliations:** 1 Institute of Biotechnology and Health, Beijing Academy of Science and Technology, Beijing 100094, China; guoyaqiong@bjast.ac.cn (Y.G.); licheng-sw@bjast.ac.cn (C.L.); chenyuexiao@bjast.ac.cn (Y.C.); zhushuai9702@163.com (S.Z.); zhicheng0408@163.com (C.Z.); 2 Institute of Chemical Biology, Shenzhen Bay Laboratory, Shenzhen 518132, China; tanminjie@szbl.ac.cn

**Keywords:** COVID-19, breastfeeding, immunoglobulin kinetics, proteomics, maternal–infant health, immune response

## Abstract

**Background:** Breast milk confers essential passive immunity to infants, particularly during viral pandemics. This study investigates dynamic changes in SARS-CoV-2-specific antibodies and proteome in the breast milk of mothers infected with either the wild-type or Omicron variants, addressing gaps in longitudinal dynamics and conserved or variant-specific immune responses. **Methods**: A prospective cohort of 22 lactating mothers infected with Omicron variant (December 2022–January 2023) was analyzed alongside a published dataset of wild-type-infected mothers (January–May 2020). Breast milk samples were collected at eight time points (1, 4, 7, 14, 21, 28, 35, 42 days post-infection) from the Omicron cohort for ELISA quantification of SARS-CoV-2-specific IgA, IgG, and IgM. Proteomic analysis was conducted for both cohorts. **Results**: Macronutrient composition remained stable throughout the post-infection period. SARS-CoV-2-specific IgA and IgG demonstrated biphasic kinetics, rapidly rising by day 14 (IgA: 0.03 to 0.13 ng/mL; IgG: 0.91 to 37.00 ng/mL) and plateauing through day 42. In contrast, IgM levels remained unchanged. Proteomic profiling identified 135 proteins associated with IgA/IgG dynamics, including variant-specific and conserved proteins. **Conclusions**: Breast milk maintains nutritional integrity while mounting robust immune responses during SARS-CoV-2 infection. These findings underscore breastfeeding as a safe and protective practice during COVID-19.

## 1. Introduction

Breast milk serves as a critical source of nutrition and immune protection for infants, particularly during the early stages of life when their immune systems are still developing [1,2]. Beyond its nutritional benefits, breast milk contains bioactive components, such as immunoglobulins, cytokines, and lactoferrin, which provide passive immunity [1,2,3]. These components plays an important role in shielding infants from infections, supporting their immune system maturation, supporting immune system maturation, and establishing the foundation for long-term health [2,4,5,6]. Maternal immunity, transferred through breast milk, is especially vital during pandemics, such as COVID-19, where infants are at increased risk of exposure to novel pathogens [7,8,9].

Among the key immune components in breast milk are immunoglobulins (IgA, IgG, and IgM) and immune proteins that act as frontline defenders against pathogens [6,10,11]. Secretory IgA, the most abundant antibody in breast milk, provides mucosal immunity by binding to pathogens and preventing their adhesion to mucosal surfaces [5,10,12,13]. IgG, though present in smaller quantities, plays a crucial role in systemic immunity and neutralization of pathogens [4,6,10]. IgM provides early-stage defense, acting as a rapid responder during acute infections [6,10]. In addition to antibodies, breast milk contains proteins with antimicrobial and antiviral properties, such as lactoferrin, lysozyme, and cytokines [3,6,11]. These proteins modulate inflammation, inhibit pathogen replication, and enhance immune responses, collectively creating a robust defense system for the infant [3,6,11]. The COVID-19 pandemic has highlighted the importance of understanding how maternal immunity is transferred through breast milk, especially in cases of natural infection [7,8,14,15]. Recent studies have demonstrated the presence of SARS-CoV-2-specific antibodies in breast milk, indicating that maternal infection induces an adaptive immune response capable of offering protection to breastfeeding infants [16,17,18]. Few studies have examined the dynamic nature of antibody levels, but those that exist are limited by small sample sizes, infrequent sampling, or a focus solely on secretory IgA (SIgA) [19,20,21]. To date, no study has comprehensively studied proteomic changes in breast milk or how they differ in response to distinct SARS-CoV-2 variants.

While SARS-CoV-2, the virus responsible for COVID-19, has undergone significant genetic evolution, leading to the emergence of multiple variants with varying levels of transmissibility, immune evasion, and pathogenicity [22,23,24,25,26], the identification of unique and conserved immune responses across variants may reveal core mechanisms critical for maternal–infant immunity. In this study, we compared wild-type and Omicron (B.1.1.529) infections to evaluate shared and variant-specific immune dynamics in breast milk. The wild-type SARS-CoV-2, identified in late 2019, represents the original wild-type virus, and formed the basis for early studies on COVID-19 transmission, immunity, and vaccine development [24,27,28,29,30]. It induced strong immune responses, including the production of antibodies, with early reports suggesting moderate neutralization capacity and persistence of antibodies in breast milk [25,26,31]. However, the emergence of new mutations in later variants raised questions about the effectiveness of immunity conferred by the wild-type strain, particularly in the context of evolving viral features [24,32]. In contrast, the Omicron variant (B.1.1.529), first identified in late 2021, is characterized by an extensive number of mutations in the spike protein, enabling it to evade immunity generated by previous infection or vaccination [24,33,34]. Omicron demonstrated higher transmissibility and caused breakthrough infections, even in vaccinated individuals, while generally inducing milder symptoms than earlier variants [25,34,35,36]. Despite these differences, conserved immune features may underpin effective maternal–infant protection. For instance, both variants likely trigger epithelial IgA responses, which is a critical mucosal defense [37,38]. Additionally, shared proteomic adaptations—such as antiviral protein induction—may sustain milk immunity across variants [39,40]. Identifying these conserved features is essential for developing universal breastfeeding guidelines, ensuring infants receive consistent protection regardless of viral evolution.

Despite growing evidence of SARS-CoV-2 antibodies in breast milk, several gaps remain in our understanding of maternal immunity. First, most existing studies have focused on vaccinated mothers [41,42,43,44,45], with limited data available on naturally infected mothers and their immune responses [16]. Secondly, the longitudinal dynamics of antibody titers and proteomic changes in breast milk during and after natural infection remain poorly characterized. Last but not least, little is known about variant-specific immune responses, including whether different SARS-CoV-2 variants induce distinct antibody profiles and proteomic shifts in breast milk. This study addresses these gaps by analyzing the dynamic changes in antibodies and proteomics in breast milk of mothers who were naturally infected with either the wild-type or Omicron variants of SARS-CoV-2.

## 2. Materials and Methods

### 2.1. Study Design and Population

Omicron cohort: The prospective cohort was carried out between December 2022 and January 2023, aligning with the last wave of the pandemic. To participate, women needed to be ≥18 years of age, lactating, have an infant less than 24 months old, and tested positive for COVID-19, or were presumptively positive during lactation. Participants were considered presumptively positive if they experienced respiratory symptoms or acute febrile illness (respiratory or other) and had contact with a qPCR confirmed or suspected case (latter prior to availability of qPCR testing). There was no limit to the amount of time from infection to enrollment; however, the infection had to occur during pregnancy or lactation. Participants were recruited from social media advertisement via Wechat (Version 8.0.57). Surveys included questions about COVID-19 testing results for mothers and infants and maternal and infant COVID-19 symptoms. COVID-19 signs and symptoms (e.g., cough, fever, congestion, fatigue, malaise, difficulty breathing, chest pain, loss of smell and/or taste, and diarrhea) were recorded at study enrollment and at each sample collection. Written informed consent was obtained from all mothers enrolled prior to the milk sample collection. The study was approved by the Nei Mongol Society of Nutrition Ethics Committee (A202212030), and the study was conducted in accordance with the Declaration of Helsinki and relevant government guidelines and regulations.

Wild-type and healthy control cohort: We utilized the raw files of proteome analysis from published literature [46]. This prospective study took place between January 2020 to May 2020. Participants were recruited from Department of Gynaecology and Obstetrics, Zhongnan Hospital of Wuhan University. Six COVID-19 infected mothers and ten healthy donors were included.

**Figure 1:**
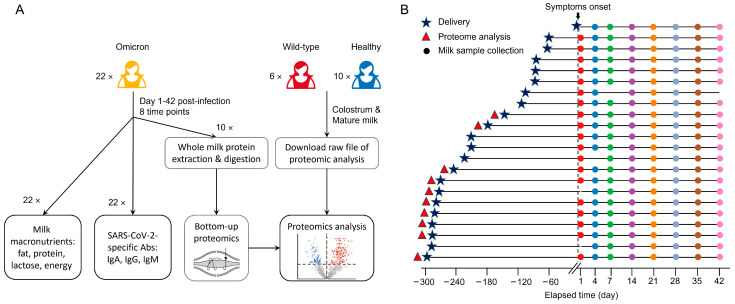
Study design. (**A**). Study workflow. (**B**). Sample collection for Omicron cohort. The day of delivery was marked by blue stars, donors for proteome analysis were marked by red triangles, the time points of milk sample collection upon symptom onset were marked by different colors of dots.

The whole workflow of this study was illustrated in Figure 1A.

### 2.2. Sample Collection

For Omicron cohort, breast milk samples were collected from 22 donors, starting the first day of the symptom onset and again at 4, 7, 14, 21, 28, 35, and 42 days post-diagnosis with different lactation days (Figure 1B). Mothers were instructed to clean the breast with a wet wipe before sample collection. Whole breast milk samples were self-collected using home-based breastmilk pumps followed by mixing and transferring into a breast milk storage bag. Samples were immediately frozen at −20 °C, transferred in dry ice within 24 h, and stored at −80 °C until thawed for analysis. The clinical characteristics of sample donors were summarized in Table 1.

### 2.3. Laboratory Analysis

#### 2.3.1. Nutritional Composition Analysis

The nutritional composition of breast milk, including macronutrients (fat, protein, carbohydrates) and energy content, was measured using a MIRIS Human Milk Analyzer (Miris AB, Uppsala, Sweden).

#### 2.3.2. SARS-CoV-2-Specific Antibodies Analysis

SARS-CoV-2-specific antibodies, including IgA, IgG, and IgM, were measured using enzyme-linked immunosorbent assays (ELISA). SARS-CoV-2-specific IgA in milk was assessed using Human anti-2019 nCoV IgA ELISA kit (My BioSource, San Diego, CA, USA). Additionally, SARS-CoV-2-specific IgG and SARS-CoV-2-specific IgM in milk were assessed using anti-SARS-CoV-2 S-RBD Human IgG or IgM ELISA kit (Proteintech, Chicago, IL, USA). Thawed milk samples were centrifuged (1000× *g*, 30 min, 4 °C) twice, and defatted supernatants were diluted with sample buffer. Results were expressed as optical density (OD) values and converted into concentration units (µg/mL) using standard curves.

#### 2.3.3. Proteomic Analysis

For the Omicron cohort, proteomic analysis was performed on samples from subjects meeting the following criteria: the first sample collection occurred ≥120 days postpartum to account for the relatively stable protein composition of mature milk; tested positive using SARS-CoV-2 antigen rapid diagnostic kits; and delivered infants were full-term infants with a gestational age of 37–40 weeks. Therefore, samples from 10 donors were chosen for proteomic analysis.

The thawed whole milk samples were digested on S-Trap™ (ProtiFi S-Trap™ Micro Spin Columns, C02-micro-80, ≤100 μg capacity; Farmingdale, NY, USA), using a modified S-Trap™ protocol adapted from the manufacturer’s guidelines and optimized based on established methodologies. The digested peptides were analyzed using liquid chromatography-tandem mass spectrometry (LC-MS/MS). Peptide separation was achieved on an EASY nLC1200 system (Thermo Fisher Scientific, San Jose, CA, USA), and analysis was performed on an Orbitrap QExactive HF mass spectrometer (Thermo Fisher Scientific, San Jose, CA, USA) in data-dependent acquisition mode and higher-energy collision dissociation (HCD) was employed. The raw data were processed with MsFragger [46] (version 22.0) against a UniProt Swiss-Prot database: Homo sapiens (August 2024; 20,435 entries) and IonQuant for protein identification and label-free quantification (LFQ). A false discovery rate (FDR) of 1% was applied, and protein abundance was normalized across samples. For further details, please refer to the Supplementary Methods section.

### 2.4. Statistical Analysis

All of the statistical analyses were performed using R version 4.3.1. Prior to selecting non-parametric methods, normality tests (the Shapiro–Wilk test) were applied when applicable. Differences between groups were analyzed using Wilcoxon rank sum test. The differences in nutritional components and antibodies were adjusted for the days post-delivery. A Natural Spline Regression Model was applied to fit the time-series data of protein abundance and antibodies, aiming to assess their consistency of trends over time. Correlation analysis was performed using Spearman’s rank correlation. In functional analysis, differential enrichment of GO terms was assessed using the hypergeometric distribution. The *p*-value < 0.05 and a fold change ≥ 2 were considered as criteria for identifying differential proteins.

All of the statistical analyses were performed using R version 4.3.1. Differences between groups were analyzed using non-parametric tests. We compared paired differences using Wilcoxon signed rank test and non-paired differences using Wilcoxon rank sum test. The differences in nutritional components and antibodies were adjusted for the days post-delivery. A Natural Spline Regression Model was applied to fit the time-series data of protein abundance and antibodies, aiming to assess their consistency of trends over time. Correlation analysis was performed using Spearman’s rank correlation. In functional analysis, differential enrichment of GO terms was assessed using the hypergeometric distribution. The *p*-value < 0.05 and a fold change >2 were considered as criteria for identifying differential proteins.

## 3. Results

### 3.1. The Macronutrient Composition of Breast Milk in the Omicron Cohort Exhibited Temporal Stability Following Infection

**Figure 2:**
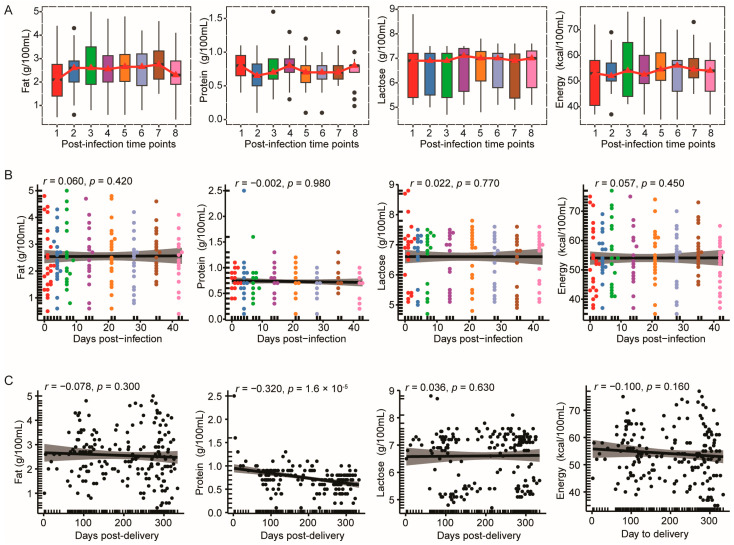
Macronutrient composition (fat, protein, lactose and energy) of breast milk in the Omicron cohort. (**A**). Boxplots of macronutrient composition in eight time points of collection. Different time points are represented in different colors. Red triangles indicate the median for each time point, which are connected by red lines. (**B**). Scatter plots and Spearman’s correlation of macronutrient composition and days post-infection. Different time points are represented in different colors. (**C**). Scatter plots and Spearman’s correlation of macronutrient composition and days post-delivery. A regression line was fitted to the data, and the 95% confidence interval for the regression line was included.

Longitudinal analyses of fat, protein, lactose, and energy content across eight post-infection time points revealed no statistically significant variations (Figure 2A). Furthermore, correlation analyses between days post-infection and macronutrient levels demonstrated no significant associations (Figure 2B). To account for the potential confounding effects of lactation stage, we investigated the relationship between postpartum duration and macronutrient profiles. Notably, protein content showed a weak but significant negative correlation with days post-delivery (*r* = −0.32, *p* = 1.6 × 10^−5^), indicating a progressive decrease over time, while fat, lactose, total solids, and energy content remained relatively stable (Figure 2C).

### 3.2. SARS-CoV-2-Specific IgA and IgG Levels Increased and Plateaued Post-Infection, While IgM Remained Stable

**Figure 3:**
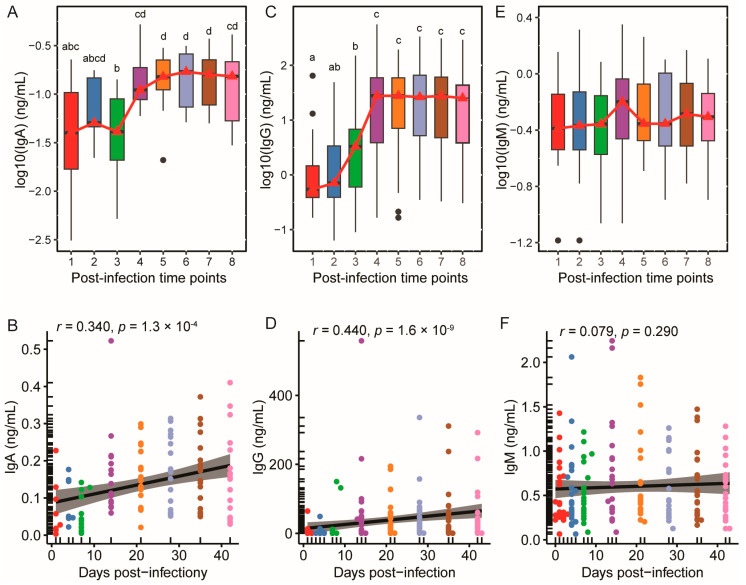
The dynamics of SARS-CoV-2-specific IgA, IgG, and IgM over post-infection. (**A**,**C**,**E**) show the log10-transformed concentrations of SARS-CoV-2-specific IgA, IgG, and IgM, respectively. Groups labeled with the same letter are not significantly different, while those with different letters indicate statistically significant differences (adjusted *p* < 0.05). Different time points are represented in different colors. Red triangles indicate the median for each time point, which are connected by red lines. (**B**,**D**,**F**) depict the scatter plots and Spearman’s correlation of SARS-CoV-2-specific IgA, IgG, and IgM and days post-delivery. Different time points are represented in different colors. A regression line was fitted to the data, and the 95% confidence interval for the regression line was included.

Following SARS-CoV-2 infection, dynamic changes were observed in the levels of SARS-CoV-2-specific immunoglobulins. As illustrated in Figure 3A, SARS-CoV-2-specific IgA levels showed a stable period from day 1 (time point 1) to day 7 (time point 3), followed by a notable upward trend from day 7 (time point 3) to day 14 (time point 4). Quantitatively, the median value of SARS-CoV-2-specific IgA surged significantly from 0.03 ng/mL to 0.13 ng/mL. Post-peak, these levels plateaued from day 21 to day 42, maintaining minimal fluctuation. The scatter plot (Figure 3B) demonstrated a significant positive correlation between SRAS-CoV-2-specific IgA and days post-infection (*r* = 0.34, *p* = 1.3 × 10^−4^).

As shown in Figure 3C, SARS-CoV-2-specific IgG levels increased significantly from day 1 (time point 1) to day 14 (time point 4), with the median rising from 0.91 ng/mL to 37.00 ng/mL. Post-day 21 (time point 5), IgG levels plateaued, maintaining stable concentrations until day 42 (time point 8). Scatter plot analysis (Figure 3D) revealed a stronger positive correlation with infection duration (*r* = 0.44, *p* = 1.6 × 10^−9^), reflecting a more pronounced time-dependent increase.

In contrast, SARS-CoV-2-specific IgM demonstrated stability across all time points. The median IgM values fluctuated slightly, with no statistically significant increase or decrease (Figure 3E). The scatter plot analysis showed no significant correlation between days post-infection and SRAR-CoV-2 specific IgM (Figure 3F). These results indicate that while IgA and IgG mounted an active, time-dependent response that plateaued post-infection, IgM levels remained consistently stable, reflecting distinct immunological dynamics among the antibody subtypes.

### 3.3. Temporal Coordination of the Breast Milk Proteome with SARS-CoV-2-Specific Antibody Dynamics

**Figure 4:**
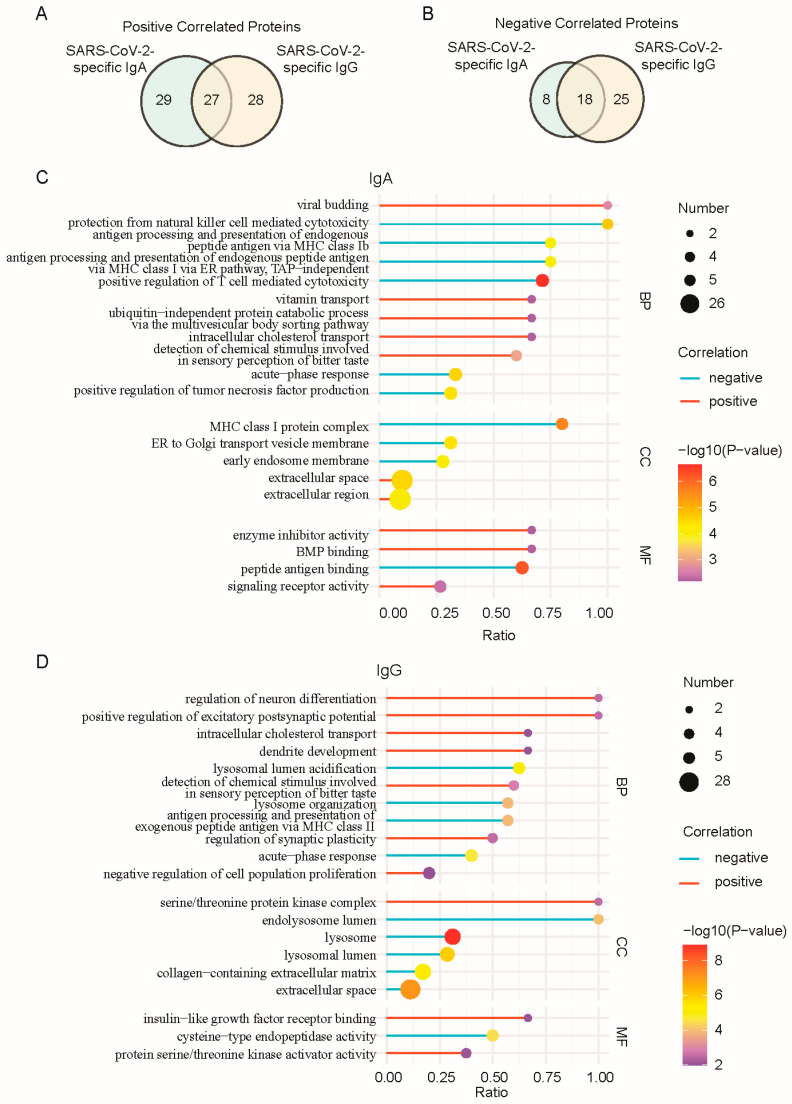
Proteins temporally coordinated with SARS-CoV-2-specific IgA and IgG kinetics. Venn diagram for proteins exhibited significant (**A**). positive correlations and (**B**), negative correlations with IgA or IgG kinetics. (**C**,**D**), respectively, represent the GO terms enriched in the differentially expressed proteins related to the changing trends of SARS-CoV-2-specific IgA and IgG. The color of the stick represents positive correlation (red) and negative correlation (blue). The color of the dot represents the *p*-value of the hypergeometric test. The size of the dot represents the number of significant correlated proteins in the corresponding GO term. BP: biological process; CC: cellular component; MF: molecular function.

As SARS-CoV-2-specific IgM was stable post-infection, the breast milk proteome was analyzed to identify proteins temporally coordinated with SARS-CoV-2-specific IgA and IgG kinetics. After filtering the dataset to retain proteins with ≥1 unique peptide and detected in >60% of samples, 972 high-confidence proteins were selected for downstream analysis (Table S1). To assess temporal associations, a Natural Spline Regression Model was applied to fit longitudinal protein abundance and antibody level trajectories, with significant correlations defined as |Pearson’s r| > 0.8 (*p* < 0.05). A total of 135 proteins exhibited significant correlations with IgA or IgG kinetics (Table S2), which correspond to approximately 14% of the milk proteome, highlighting the plasticity of the lactating immune system in response to SARS-CoV-2 infection. As shown in Figure 4A,B, for IgA, 56 proteins showed positive correlations, while 26 were negatively correlated. Similarly, 55 proteins were positively correlated with IgG, and 43 were negatively correlated. Among these, 27 proteins were positively correlated with both antibodies, and 18 were negatively correlated.

Notably, immunoglobulin fragments (e.g., light chains, variable regions) were prominently enriched among positively correlated proteins, indicating that elevated SARS-CoV-2-specific IgA and IgG levels corresponded with increased proteolytic fragments during antibody secretion or turnover. As depicted in Figure 4C and Table S3, The Gene Ontology (GO) analysis of proteins positively correlated with SARS-CoV-2-specific IgA revealed enrichment in biological process (BP) terms, including viral budding, intracellular cholesterol transport, and detection of chemical stimulus involved in sensory perception of bitter taste. At the molecular function (MF) level, these proteins clustered in enzyme inhibitor activity, BMP binding, and signaling receptor activity. SARS-CoV-2-specific IgG was positively correlated with proteins enriched in BP terms such as neuron differentiation, intracellular cholesterol transport, and sensory perception of bitter taste (Figure 4D and Table S4). In the cellular component (CC) category, serine/threonine protein kinase complex was overrepresented, while MF enrichment included insulin-like growth factor receptor binding and protein serine/threonine kinase activator activity. Although numerous positively correlated immunoglobulin proteins were not included in GO analysis, their intrinsic association with adaptive immunity warranted attention.

Conversely, SARS-CoV-2-specific IgA were negatively correlated with proteins enriched in biological processes such as natural killer cell-mediated cytotoxicity, MHC class Ib-mediated antigen presentation, and T cell-mediated cytotoxicity (Figure 4C). The CC category highlighted MHC class I protein complex, emphasizing their structural roles in antigen presentation. For SARS-CoV-2-specific IgG (Figure 4D), negatively correlated proteins showed significant BP enrichment in antigen processing and presentation of exogenous peptide antigen via MHC class II and acute-phase response. In the CC category, lysosome lumen and extracellular space were prominently enriched, while peptide antigen binding emerged as a key MF term. Notably, both IgA- and IgG-associated negatively correlated proteins were enriched in terms related to antigen processing and presentation via MHC classes (BP) and acute-phase response (BP). This collectively reflects a temporal transition from early cell-mediated innate responses to late adaptive humoral immunity, illustrating a dynamic balance between innate antigen presentation and adaptive antibody-driven mechanisms in sustaining antiviral protection.

### 3.4. Proteomic Analysis Revealed 24 Proteins with Consistent Change Trends Between Wild-Type and Omicron Variants

The wild-type SARS-CoV-2 milk proteome was analyzed using the same criteria as the Omicron cohort: proteins with ≥1 unique peptide and detection in >60% of samples. This yielded 967 high-confidence proteins (Table S5). To identify conserved and variant-specific proteomic responses, we first performed differential expression analysis on wild-type colostrum (COVID-19_colostrum vs. COVID-19_mature milk) using rigorous statistical thresholds (fold change ≥ 2, adjusted *p* < 0.05). These proteins were subsequently compared to the Omicron-derived proteomic signatures significantly correlated with SARS-CoV-2-specific IgA/IgG kinetics. This integrative approach revealed 13 proteins exhibited variant-specific changes (Figure S1) and 24 proteins with conserved abundance trends between wild-type and Omicron infections (Figure S2).

**Figure 5:**
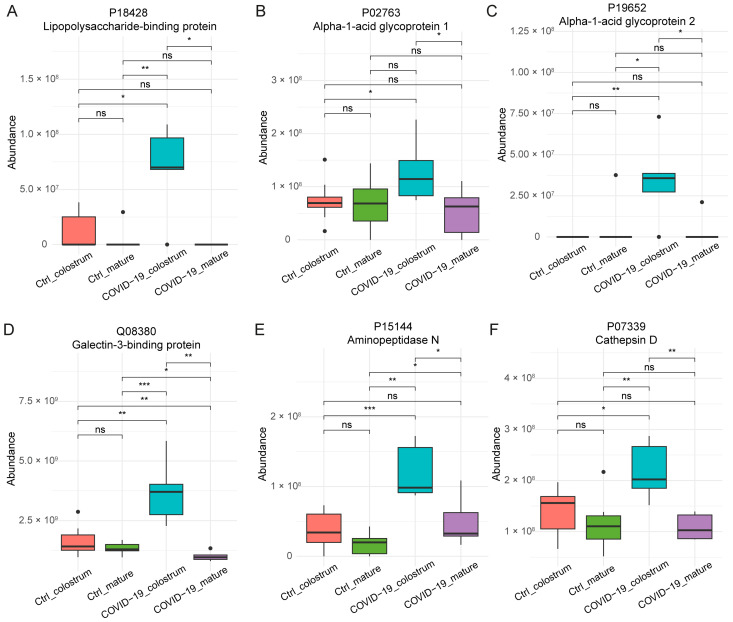
Box plots of abundance of selected proteins exhibiting conserved expression trends across wild-type and Omicron variants. (**A**). Lipopolysaccharide-binding protein (LBP). (**B**). Alpha-1-acid glycoprotein 1 (AGP1). (**C**). Alpha-1-acid glycoprotein 2 (AGP2). (**D**). Galectin-3-binding protein (Gal-3BP). (**E**). Aminopeptidase N (APN). (**F**). Cathepsin D. The *x*-axis categorizes samples into four groups: Ctrl_colostrum (healthy control colostrum) is represented in pink, Ctrl_mature (healthy control mature milk) is represented in green, COVID-19_colostrum (COVID-19 colostrum) is represented in blue, and COVID-19_mature (COVID-19 mature milk) is represented in purple. The *y*-axis represents protein abundance. Statistical significance between groups is denoted by: ns (not significant), *p* ≥ 0.05; * p < 0.05; ** p < 0.01; *** p < 0.001.

Variant-specific proteins were dominated by immunoglobulins, particularly J chain, heavy mu, and variable chains, indicating the adaptive immune responses tailored to viral genetic diversity. In contrast, conserved proteins across wild-type and Omicron infections included several acute-phase response proteins, such as lipopolysaccharide-binding protein (LBP, Figure 5A), alpha-1-acid glycoprotein (AGP, Figure 5B,C), and Galectin-3-binding protein (Gal-3BP, Figure 5D). These proteins are integral to innate immune activation, bacterial recognition, and inflammation modulation. Protease/peptidase enzymes like aminopeptidase N (APN, Figure 5E) and Cathepsin D (Figure 5F) were also conserved. Collectively, these conserved proteins reflect invariant early innate immune responses critical for viral infection, as observed in both wild-type and Omicron variants.

## 4. Discussion

### 4.1. Main Findings and Comparison with Previous Studies

This study provides a comprehensive analysis of the immune responses and proteomic adaptations of breast milk in mothers infected with either wild-type or Omicron SARS-CoV-2 variants. Collectively, our study revealed three major findings: (1) Macronutrient composition remained stable post-infection, ensuring nutritional continuity for infants despite maternal illness; (2) SARS-CoV-2-specific IgA and IgG exhibited biphasic kinetics with prolonged plateau phases, indicating sustained antiviral immunity in breast milk; and (3) proteomic signatures reflected a transition from early cell-mediated to late humoral immunity, with 24 proteins showing conserved responses across variants and 13 proteins with variant-specific response. These results underscore the lactating immune system’s ability to dynamically adapt to viral challenges while preserving nutritional quality, offering critical evidence for breastfeeding guidance during pandemics. The identification of invariant proteomic responses across variants highlights core molecular mechanisms of milk immunity, which may inform strategies to enhance passive protection for infants.

The impact of maternal viral infection on breast milk macronutrient composition remains an area of interest, particularly in the context of acute infections. Previous studies have focused primarily on chronic viral infections. For instance, studies in women living with human immunodeficiency virus-1 (HIV-1) have reported altered milk composition, including increased protein and possibly lower carbohydrate [47,48]. Similarly, hepatitis B virus (HBV) infection has been associated with elevated levels of glucose and albumin [49]. Fewer studies have addressed the effects of acute viral infections, like SARS-CoV-2, on breast milk composition. In our study, we observed no significant changes in breast milk macronutrients (fat, protein, lactose, and energy content) over 42 days following Omicron infection. This finding contrasts with a previous cross-sectional study, which reported increased fat and lactose content in the milk of vaccinated and COVID-19-infected mothers [50]. However, discrepancies may be attributed to methodological differences, including the absence of lactation stage data and lack of longitudinal follow-up. These inconsistencies highlight the need for standardized, longitudinal studies to clarify the impact of maternal acute infection on human milk composition.

Numerous studies have documented the presence of SARS-CoV-2-specific antibodies in breast milk following infection [16,20,21,51], but few have captured the early post-infection window or followed antibody responses longitudinally. In our Omicron-infected cohort, serial sampling over 42 days revealed biphasic dynamics for SARS-CoV-2-specific IgA and IgG: rapid induction within 14 days post-infection, followed by stable plateau phases through day 42. This pattern contrasts with vaccine-induced antibody responses. Most studies evaluating mRNA vaccine responses in lactating individuals have reported a less dominant IgA response [21,52,53,54], and a delayed but robust milk IgG response, typically emerging 14–21 days after the first dose, with a sharp increase peaking around 7 days after the second dose, and remaining elevated for 4–6 weeks [44,55,56,57,58]. These findings emphasize the difference in immune responses between infection and vaccination and highlight the importance of longitudinal studies to fully characterize antibody dynamics in lactating mothers post-infection. In contrast to the dynamic IgA and IgG responses, SARS-CoV-2-specific IgM levels remained stable throughout the 42-day observation period. This finding aligns with previous reports demonstrating divergent IgM dynamics compared to IgG [59]. Unlike IgG, which is transferred from blood, or IgA, which shows dynamic mucosal boosting, IgM is produced predominantly in mucosa-associated lymphoid tissue within the mammary gland. This may reflect limited class-switch recombination and the distinct differentiation trajectory of IgM-secreting B cells residing in the lactating breast.

Notably, these breast milk antibody dynamics also differ from serum antibody profiles reported in COVID-19 patients. In serum, IgA levels in severe cases surged to peak concentrations by day 11, whereas mild cases showed delayed induction with sustained increases through week 3 [60]. IgG seroconversion rates reached 100% in severe cases by week 3, compared to 68.9% in mild cases, with both groups showing rising IgG trends [60]. Another study demonstrated that SARS-CoV-2-specific IgA and IgG in breast milk were detected several weeks post-symptom onset and exhibited robust neutralizing activity against viral variants [31]. These findings corroborate our observation of prolonged antibody persistence in breast milk and highlight the compartmentalized immune responses between systemic and mucosal (breast milk) compartments, underscoring the unique immunological microenvironment of the mammary gland during infection.

Proteomic analysis identified 135 proteins (14% of the dataset) associated with IgA/IgG kinetics. Positively correlated proteins were enriched in immunoglobulins and their fragments, including light chains and variable regions, indicating active antibody secretion or turnover. These fragments, co-expressed with SARS-CoV-2-specific antibodies, may serve as biomarkers of immune activation. Additionally, proteins clustered in viral budding and cholesterol transport, suggesting interactions with the viral envelope, potentially interfering with the release of infectious virions [61]. Another enriched GO term was sensory perception of bitter taste, which may be linked to the immune response in the mammary gland. Given that dysgeusia (taste alteration) is a common symptom of COVID-19 [62], and bitter taste receptors are expressed in mucosal tissues, including the sinonasal epithelium and mammary gland, where they play roles in chemosensation and innate immunity. These receptors’ activation may trigger immune responses that contribute to the infant’s protection via breastfeeding, with their activity potentially influencing the clinical course and symptom duration of SARS-CoV-2 infection [63].

Negatively correlated proteins with IgA/IgG included antigen presentation machinery (e.g., HLA I and HLA II), which declined as antibodies rose. Meanwhile, several innate immunity related terms also showed negative correlation with the SARS-CoV-2-specific antibodies, such as acute-phase response and protection from natural killer cell mediated cytotoxicity. This shift reflects a transition from early cell-mediated innate immunity to late humoral immunity (antibody dominance), a hallmark of resolving viral infections. Similar findings have been reported in other studies, where the decline of acute-phase proteins (e.g., C-reactive protein) coincided with the activation of antibody responses during infection resolution [64]. The inverse relationship between HLA molecules and IgG further supports this transition, as HLA are key in antigen presentation to T cells and the initiation of cellular immunity [65]. GO analysis of negatively correlated proteins revealed enrichment in pathways like antigen processing via MHC class I/II and acute-phase response, underscoring their role in early immune surveillance.

Thirteen variant-specific proteins were identified between the wild-type and Omicron variants, which were dominated by immunoglobulins, particularly J chain (IGJ), heavy mu chain (IGHM), and variable chains (IGHV/IGLV families). These immunoglobulins likely represent adaptive immune responses tailored to variant-specific spike mutations, as B cell receptor diversification is driven by antigenic evolution [66]. In contrast, twenty-four proteins showed conserved abundance changes across wild-type and Omicron infections, including acute-phase proteins such as lipopolysaccharide-binding protein (LBP), alpha-1-acid glycoprotein (AGP), and Galectin-3-binding protein (Gal-3BP). LBP, a key acute-phase reactant, is elevated in sepsis and serves as a biomarker of intestinal barrier dysfunction [67]. In COVID-19, plasma LBP correlates with inflammasome activation and gut permeability, suggesting a role in systemic inflammation [68]. AGP, another acute-phase protein, is upregulated in severe COVID-19, and correlates with IL-6 and C-reactive protein levels, underscoring its involvement in cytokine storm pathogenesis [69]. Similarly, Gal-3BP promotes IL-6 secretion by activating Galectin-3, linking it to pro-inflammatory signaling [70]. Conserved proteases like aminopeptidase N (APN) and Cathepsin D highlight shared viral interaction mechanisms. APN, a known entry receptor for porcine delta coronavirus, may facilitate SARS-CoV-2 attachment to host cells [71]. Cathepsin D, a lysosomal protease, mediates viral spike protein priming during cell entry [72]. Collectively, these conserved proteins reflect invariant early innate immune responses critical for viral recognition and clearance across variants. Notably, these proteins were altered in colostrum, but normalized in mature milk, indicating transient adaptations during the acute phase of infection. This temporal pattern may reflect a prioritization of immune protection in early lactation, aligning with the critical role of colostrum in neonatal immunity.

### 4.2. Strengths and Limitations

A key strength of this study is its longitudinal design, with dense sampling across 42 days post-infection, which allows for a detailed and dynamic characterization of antibody kinetics in breast milk following SARS-CoV-2 infection. This comprehensive timeline enables the capture of both early- and late-phase immune responses. Furthermore, this study combines quantitative antibody assays with proteomic profiling, offering a system-level understanding of milk immunity. By integrating both antibody responses and proteomic changes, we were able to capture key immune proteins that are dynamically regulated in response to infection, shedding light on the molecular mechanisms underpinning lactational immunity. This dual approach allows for a deeper understanding of how immune proteins and antibodies interact, creating a more comprehensive picture of maternal immune protection transferred through breastfeeding. The inclusion of wild-type SARS-CoV-2 data from previously published literature allows for valuable comparative analysis between variants, providing insights into the variant-specific immune responses that may influence antibody dynamics and milk proteomics.

This study has several limitations that should be considered. First, the Omicron cohort included only 22 donors, which may limit the overall statistical power of the study and the generalizability of the findings. Second, the wild-type SARS-CoV-2 data were derived from previously published literature, which may introduce batch effects or variations in experimental protocols that could influence the comparability of results. Third, while the proteomic correlations identified in this study provide valuable insights into the dynamic changes in milk immunity, the functional significance of these correlations remains speculative. These findings would benefit from further validation through in vitro studies to establish mechanistic pathways and causality.

### 4.3. Clinical Implications and Future Directions

It has been widely reported that the risk of direct SARS-CoV-2 transmission from mother to infant through breastfeeding or close proximity is low, especially when appropriate hygiene measures are taken. The preservation of both macronutrient content and robust antibody responses in breast milk during acute SARS-CoV-2 infection underscores the safety and immunological value of continued breastfeeding. The sustained presence of SARS-CoV-2-specific IgA and IgG suggests ongoing mucosal protection for infants, which may help reduce viral transmission risk or disease severity. The detection of conserved proteomic signatures across variants also indicates that breast milk retains a core set of antiviral defense mechanisms, regardless of viral strain. These findings support current public health recommendations encouraging breastfeeding during maternal COVID-19 illness, even amid evolving variants.

Future research should focus on larger, variant-specific cohorts to validate these findings and explore potential severity-dependent differences in milk immune responses. Mechanistic studies using in vitro or ex vivo models are needed to determine the functional significance of specific immunoglobulin fragments and antigen presentation proteins identified in this study. Extending follow-up beyond 42 days would also help characterize long-term persistence and transitions in milk immunity. Critically, studies linking maternal milk profiles to infant health outcomes—such as infection rates, vaccine responsiveness, and mucosal immunity—will be essential to understand the translational significance of breastfeeding during maternal viral infection.

## 5. Conclusions

This study provides novel insights into the dynamic interplay between antibodies and the breast milk proteome during SARS-CoV-2 infection. The stability of macronutrients, biphasic antibody responses, and proteomic transitions highlight the lactating immune system’s resilience and adaptability. These findings contribute to our understanding of maternal–infant immunity during pandemics and inform strategies to optimize breastfeeding guidance for COVID-19-positive mothers.

## Figures and Tables

**Table 1:** Clinical characteristics of sample donors.

Characteristics	Mothers	Infants
Age (years)	32.50 ± 3.59	2~334 (days)
Weight (kg)	62.41 ± 11.32	NA
BMI	24.02 ± 3.87	NA
Gestational Week, *n* (%)		
37–40 weeks	17 (77.27)	NA
≤37 weeks	1 (4.55)	NA
≥40 weeks	4 (18.18)	NA
Gravidity, *n* (%)		
First	17 (77.27)	NA
Second	5 (22.73)	NA
Symptom, *n* (%)		
Fever	13 (59.09)	14 (63.64)
Headache or muscle aches	16 (72.73)	7 (31.82)
Sore throat	16 (72.73)	7 (31.82)
Runny nose	18 (81.82)	8 (36.36)
Cough	21 (95.45)	10 (45.45)
Loss of appetite	8 (36.36)	10 (45.45)
Diarrhea	6 (27.27)	2 (9.09)

Data are presented as mean (±SD) for continuous variables and total number (percentage) for categorical variables. NA indicates not applicable.

## Data Availability

The proteome data (dataset identifier PXD062335) have been deposited to the ProteomeXchange Consortium (http://proteomecentral.proteomexchange.org; last accessed on 24 March 2025) via the iProX partner repository PRIDE [74].

## Supplementary Materials

The following supporting information can be downloaded at: https://www.mdpi.com/article/10.3390/nu17081396/s1, Figure S1. Proteins with distinct abundance trends between wild-type and Omicron infections. Statistical significance between groups is denoted by: ns (not significant), *p* ≥ 0.05; * *p* < 0.05; ** *p* < 0.01; *** *p* < 0.001. Figure S2. Proteins with conserved abundance trends between wild-type and Omicron infections. Statistical significance between groups is denoted by: ns (not significant), *p* ≥ 0.05; * *p* < 0.05; ** *p* < 0.01; *** *p* < 0.001; **** *p* < 0.0001. Table S1. Breast milk proteome in mothers infected by Omicron variant. Table S2. Proteins exhibited significant correlations with SARS-CoV-2-specific IgA or IgG kinetics. Table S3. GO analysis results for proteins temporally coordinated with SARS-CoV-2-specific IgA kinetics. Table S4. GO analysis results for proteins temporally coordinated with SARS-CoV-2-specific IgG kinetics. Table S5. Breast milk proteome in mothers infected by wild-type variant. References [46,73] are cited in Supplementary Materials.
