# Supplementary material for: Dynamic Changes in Antibodies and Proteome in Breast Milk of Mothers Infected with Wild-Type SARS-CoV-2 and Omicron: A Longitudinal Study
Source: Nutrients. 2025 Apr 21;17(8):1396. doi: 10.3390/nu17081396 (PMC12030011; doi:10.3390/nu17081396)
Supplement: Supplementary file 1 [file nutrients-17-01396-s001.zip › Supplementary Methods.pdf]

Supplementary Methods:

Milk Sample Preparation for Proteomic Analysis

A 5 µL milk sample was added to 45 µL of MilliQ water, followed by adding SDS (final concentration 5%) (Amresco, Solon, OH, USA) and 50 mM triethylammonium bicarbonate buffer (TEAB) (Sigma-Aldrich, St. Louis, MO, USA). Subsequently, tris (2-carboxyethyl) phosphine hydrochloride (TCEP) (Invitrogen, Carlsbad, CA, USA) was added to a final concentration of 10 mM, and the mixture was incubated at 55 °C for 30 min. After cooling to room temperature, chloracetamide (CAA) (Sigma-Aldrich, St. Louis, MO, USA) was added to reach a final concentration of 80 mM and incubated for 30 min. Then, 12% phosphoric acid (PA) (Sigma-Aldrich, St. Louis, MO, USA) was added to a final concentration of 1.1% to adjust the pH to less than 1. Next, 165 µL of wash buffer (90% MeOH/100 mM TEAB, pH 7.55) was added, and the sample was transferred to an S - Trap and centrifuged at 4000×g for 30 s. The washing step was repeated four times by adding 150 µL of wash buffer each time and centrifuging at 4000×g for 30 s. After that, a final centrifugation at 4000×g for 1 min was carried out to completely remove the wash buffer. Trypsin (Promega, Madison, WI, USA) was added at a 1:10 (w:w) ratio (dissolved in 125 µL of 50 mM TEAB digestion buffer), and the sample was digested at 47 °C for 1 h. For elution, three different buffers were used. First, 40 µL of 50 mM TEAB was added and centrifuged at 4000×g for 1 min. Then, 40 µL of 0.2% formic acid (Fisher Scientific, Pittsburgh, PA, USA) was added and centrifuged at 4000×g for 1 min. Finally, 40 µL of 50% acetonitrile (Fisher Scientific, Pittsburgh, PA, USA) was added and centrifuged at 4000×g for 1 min. The elutes were combined and concentrated under a spin vacuum and stored in -80°C.

Liquid Chromatography-Tandem Mass Spectrometry

Prior to the liquid chromatography (LC)–tandem MS (MS/MS) analysis, the dried tryptic peptides were reconstituted in 0.1% formic acid. Subsequently, peptide separation was carried out using an EASY nLC1200 (Thermo Fisher Scientific, San Jose, CA, USA) with an in-house packed trap column (150µm I.D. × 1cm) with 1.9 µm C18 reverse-phase fused-silica (Michrom Bioresources, Inc., Auburn, CA, USA) and capillary column (150µm I.D. × 20cm) with the same material as trap column. The mobile - phase solvents were composed as follows: Solvent A was 0.1% formic acid in water, while solvent B was 0.1% fomic acid in 80% acetonitrile. During the trapping process, a flow rate of 600 nL/min was maintained for 5 minutes with 3% of solvent B. For the separation step, a flow of 600 nL/min was applied over a 120-minute period. The gradient of solvent B was carefully adjusted: it increased from 6% to 12% over 8 min, 12% to 30% B over 82 min, 30% to 40% B over 20min, 40% to 95% over 2min, 95% B for 8min, and 95% to 30% B over 2 min. The eluted peptides were subjected to electrospray ionization at a spray voltage of 2.0 kV and were then analyzed using an Orbitrap QExactive HF mass spectrometer (Thermo Fisher Scientific, San Jose, CA, USA) that was coupled to the LC system.

The MS analyses were conducted in data-dependent acquisition (DDA) mode. Higher-energy collision dissociation (HCD) was employed for MS/MS fragmentation to obtain detailed peptide fragmentation information. The MS1 scan range was 400–1650 m/z with a 60,000 resolution at 200 m/z, and the automated gain control (AGC) was set at 3 × 106 with a maximum injection time of 80 ms. With charge-states screening enabled, precursor ions in 2+ to 6+ charge states with intensities > 2 × 104 were selected for MS/MS (isolation window: 1.6 m/z) followed by HCD

dissociation with a normalized collision energy of 27% and Orbitrap detection with a fixed first mass of 120 m/z, a 30,000 resolution (at 200 m/z), and an AGC of 2 × 104 with a 25 ms maximum injection time. A 30 s dynamic exclusion was set. The mass spectrometer was operated in DDA mode with a top 20 loop.

The raw data obtained from shotgun LC-MS/MS data of wild-type [1] and Omicron were searched with MsFragger [2] (version 22.0) against a UniProt Swiss-Prot database: Homo sapiens (August 2024; 20,435 entries). For identification, cysteine (Cys) carbamidomethylation was set as a fixed modification, while methionine (Met) oxidation and N-terminal acetylation were considered as variable modifications. Trypsin was specified as the cleavage enzyme, with a maximum of two missed cleavages permitted. A precursor mass tolerance of 20 ppm and a fragment mass tolerance of 20 ppm were applied and a false discovery rate (FDR) of 1% was set. For label-free quantification (LFQ), IonQuant was employed. To enhance the reliability of quantification, the "match between runs" feature was enabled within a time window of 1 minute, and an FDR of 1% was maintained. Additionally, intensity normalization across different runs was carried out. The intensities of the extracted ion chromatograms at the MS1 level were utilized for the LFQ of peptides. The abundance of each identified protein was determined based on the intensities of its unique peptides. Each protein's relative abundance was defined as the ratio of its LFQ intensity to the sum of all protein intensities, ensuring normalization across samples.

1. Guo, J., et al., *Proteomic Analysis of Human Milk Reveals Nutritional and Immune Benefits in the Colostrum from Mothers with COVID-19.* Nutrients, 2022. **14**(12): p. 2513.
2. Polasky, D.A., et al., *MSFragger-Labile: A Flexible Method to Improve Labile PTM Analysis in Proteomics.* Mol Cell Proteomics, 2023. **22**(5): p. 100538.
